# Intentional Recreational Abuse of Quetiapine Compared to Other Second-generation Antipsychotics

**DOI:** 10.5811/westjem.2016.10.32322

**Published:** 2016-12-06

**Authors:** Lauren Klein, Stacey Bangh, Jon B. Cole

**Affiliations:** * Hennepin County Medical Center, Department of Emergency Medicine, Minneapolis, Minnesota; † Minnesota Poison Control System, Minneapolis, Minnesota

## Abstract

**Introduction:**

Case reports and poison center data have demonstrated that the second-generation antipsychotic quetiapine is being obtained and used for recreational abuse. The purpose of this study was to describe the relative rates of single-substance abuse for different atypical antipsychotics and compare their demographic and clinical features.

**Methods:**

We conducted a 10-year retrospective analysis of the National Poison Data System (NPDS) database (2003 – 2013). Trained nurses and pharmacists with specialty training in toxicology prospectively collect all NPDS data at poison control centers around the United States. We queried the NPDS for all cases of single-substance second-generation antipsychotic exposures coded as “intentional abuse.” The data provided by the NPDS regarding rates and clinical features of quetiapine abuse and the abuse of all other second-generation antipsychotics were compared and described descriptively.

**Results:**

During the study period, 2,118 cases of quetiapine abuse and 1,379 cases of other second-generation antipsychotic abuse were identified. Quetiapine abuse was more common than the abuse of other second-generation antipsychotics, compromising 60.6% of all abuse cases during the study period. After quetiapine, the next most frequently abused medications were risperidone (530 cases, 15.2%) and olanzapine (246 cases, 7.0%). For all second-generation antipsychotics including quetiapine, central nervous system clinical effects were most common, including drowsiness, confusion, and agitation. Other serious clinical effects observed with second-generation antipsychotic abuse included hypotension, respiratory depression, and seizures.

**Conclusion:**

Quetiapine abuse is relatively common, and is abused far more often than any other second-generation antipsychotic. Emergency physicians should be aware of the clinical effects that may occur after second-generation antipsychotic abuse.

## INTRODUCTION

Quetiapine is a second-generation antipsychotic (SGA) approved for use in schizophrenia and bipolar disorder.^1^ It is also commonly prescribed for generalized anxiety disorder, major depression, and mood disorders.^2^,^3^ While the majority of quetiapine prescriptions are used for their intended purpose, some patients obtain quetiapine from both legitimate and illicit sources and use this medication as a drug of abuse.

Although SGAs are not classically considered to have significant abuse potential, over the last decade case reports and poison center data have demonstrated that quetiapine abuse is a common phenomenon.^4^–^15^ The intentional abuse of quetiapine reportedly achieves a variety of desirable recreational alterations of sensorium, including anxiolysis, hypnosis, and euphoria.^4^,^5^,^14^,^16^,^17^ Quetiapine is also abused concomitantly with other illicit substances, such as cocaine or other sympathomimetics, to enhance their effects or to aid in self-treatment of withdrawal.^8^,^16^

Quetiapine abuse is particularly concerning given the morbidity and mortality associated with its “non-prescribed” use. This has been demonstrated most extensively in the literature discussing quetiapine overdoses. Many studies have shown that patients who overdose on quetiapine are at risk for coma, hypotension, respiratory depression, seizure, and death.^18^–^22^ Additional literature demonstrates the need for advanced resuscitative measures after quetiapine overdose, including intravenous fat emulsion (Intralipid®) therapy and extracorporeal membrane oxygenation.^23^,^24^ Based on these observations, some have remarked that quetiapine ingestions may be more dangerous than comparable ingestions of other antipsychotics.^18^

The purpose of this study was to identify the relative incidence of intentional recreational single-substance abuse of quetiapine compared to other SGAs, and to compare their demographic and clinical features. This study question is of great importance because if quetiapine abuse is in fact as common as prior literature suggests, quetiapine abuse presenting to the emergency department (ED) should be better characterized to prepare emergency physicians for management of these patients.

## METHODS

### Study Setting

This study is a retrospective review evaluating the intentional recreational abuse of quetiapine compared to other SGAs reported to the National Poison Data System (NPDS) from September 1, 2003, to September 1, 2013. Approval for this study was obtained from the institutional review board human subjects research committee.

The NPDS is owned and managed by the American Association of Poison Control Centers (AAPCC); it contains over 62 million exposure cases on over 420,000 different products since 1983. Nurses and pharmacists with specialty training in toxicology collect all NPDS data in real time. These trained experts use a systematic tool to assign clinical effects, clinical outcomes, and reasons for exposure to each case in a prospective manner. The NPDS also obtains close follow up by communicating directly with the caregivers for each case.

### Definitions

The definition of “intentional abuse” used by AAPC-accredited poison centers is “an exposure resulting from the intentional improper or incorrect use of a substance where the patient was likely attempting to gain a high, euphoric effect or some other psychotropic effect, including recreational use of a substance for any effect.”^25^ In terms of NPDS coding, intentional abuse is a distinct entity from “intentional misuse” (“an exposure resulting from the intentional improper or incorrect use of a substance for reasons other than the pursuit of a psychotropic effect”) and “intentional – suspected suicidal” (“an exposure resulting from the inappropriate use of a substance for self-harm or for self-destructive or manipulative reasons”).^25^

The AAPCC also designates clinical outcomes for each individual case. Again, these outcomes are determined using standardized criteria.^26^ Table 1 defines criteria for each clinical outcome from the NPDS guidelines, and examples may be found in the NPDS coding manual.^25^

### Study Protocol

We queried the NPDS for all SGA exposures coded as “intentional abuse.” Only single-substance exposures (those without co-ingestions of other substances) were included. We identified cases by the NPDS using all known product codes (generic and brand names for all formulations). The SGAs included in the query were quetiapine (Seroquel©), risperidone (Risperdal©), clozapine (Clozaril©), olanzapine (Zyprexa©), iloperidone (Fanapt©), arirpiprazole (Abilify©), paliperidone (Invega©), ziprasadone (Geodon©), asenapine (Saphris©), and lurasidone (Latuda©). We did not include combination formulations with drugs from other classes. Exclusion criteria were cases coded as “confirmed non-exposure,” as well as cases where the patient age was less than 10 years old, as these were unlikely to be intentional abuse.

After acquisition of the electronic NPDS database, we divided cases into study cohorts. The primary study cohort included all cases of quetiapine abuse. Additional study cohorts for comparison included a group of all other SGA cases combined, in addition to cohorts of each individual SGA. If an individual SGA had fewer than 50 total cases reported to the NPDS over the 10-year period, it was excluded from comparative analysis as an individual cohort, but was still included in the cohort of all other antipsychotics combined.

For the first part of the investigation, we analyzed demographic data. The data points extracted included age, gender, route of exposure, chronicity of abuse, and patient disposition. This demographic analysis included cases with any medical outcome (no effect, minor effect, moderate effect, major effect, death, unable to follow, and not followed). The next part of the investigation sought to describe the clinical features of SGA abuse. This part of the analysis only included cases with known outcomes (no effect, minor effect, moderate effect, major effect, or death). This was done to improve the accuracy of the reported clinical data. The data collected regarding clinical features of SGA abuse included data on clinical effects (agitated/irritable, ataxia, coma, confusion, dizziness/vertigo, drowsy/lethargy, dystonia, hallucinations, seizure, slurred speech, conduction disturbance, dysrhythmia, electrocardiogram changes, hypotension, syncope, tachycardia, elevated creatine kinase/rhabdomyolysis, respiratory arrest, respiratory depression), therapies provided (alkalinization, benzodiazepines, cathartics, charcoal, CPR, intravenous fluids, intubation, lavage, naloxone, oxygen, physostigmine, sedation, vasopressors), and medical outcome (no effect, minor effect, moderate effect, major effect, or death).

### Data Analysis

All data were obtained directly from the electronic NPDS database and analyzed with descriptive statistics. For all variables previously mentioned, we determined proportions for each cohort (quetiapine cohort, all other SGAs combined cohort, and each individual SGA cohort). All analyses were conducted using STATA (Version 12.1, StataCorp, College Station, TX).

## RESULTS

During the study period there were 2,134 total cases of quetiapine exposures and 1,398 cases of SGA exposures coded as intentional abuse reported to the NPDS. In the quetiapine cohort, 16 cases were excluded (six due to age less than 10 years, 10 confirmed non-exposures), leaving 2,118 for analysis. In the other SGA cohort, we excluded 19 cases (17 due to age less than 10 years, two confirmed non-exposures), leaving 1,379 for analysis.

Quetiapine was the most commonly abused SGA (n = 2118) during the study period, accounting for 60.6% of all cases. The next most frequently abused SGA was risperidone (530), followed by olanzapine (246), aripiprazole (229), ziprasadone (229), clozapine (101), paliperidone (34), asenapine (6), iloperidone (2), and lurasidone (2). Table 2 depicts demographic data. Table 3 depicts patient disposition.

Of the 2,118 cases in the quetiapine cohort, there were 1,446 cases with known outcomes. Of the 1,379 cases in the cohort of all other SGAs, there were 919 with known outcomes. Table 4 demonstrates these medical outcomes for each cohort. Table 5 describes the clinical effects seen with SGA intentional abuse. Table 6 describes the therapies provided for each cohort.

## DISCUSSION

Emergency physicians encounter substance abuse on a daily basis. Although quetiapine has not classically been considered a “drug of abuse,” in this last decade there have been many reports in the medical literature as well as in the media describing this phenomenon.^4^, ^5^, ^7^–^9^, ^14^, ^15^ Emergency medicine literature has previously been far more robust in describing the clinical features and adverse events associated with quetiapine overdoses;^18^–^23^ however, recreational abuse of quetiapine appears to be another significant public health problem that emergency physicians must be aware of.

This study corroborates that quetiapine is the most commonly abused SGA. Although perspectives from case reports^4^,^14^, and survey data^16^ suggested this was likely to be the case, this NPDS query confirmed that quetiapine abuse was identified and prospectively reported more frequently than any other SGA; in fact, quetiapine was abused more often than all other SGAs combined. In addition to our work, the most comprehensive publication thus far supporting this notion was a study using the Drug Abuse Warning Network (DAWN). 27 The DAWN is a public health surveillance system in the United States that uses medical record data from a representative group of hospitals in addition to population data to approximate prevalence. This differs from the NPDS dataset in that the data from the DAWN is estimated based on retrospective chart review, rather than prospectively identified cases called into national poison centers. Despite these different methods, the authors of this study found similar results; quetiapine-related ED visits increased by 90% from 2005 to 2011, including visits for misuse/abuse, suicide, and adverse events. Although they did combine visits for misuse and abuse, they identified 27,114 visits for these purposes during their study period, of which 6,780 were single-substance (quetiapine-only) visits. This number represented 52% of all SGA misuse/abuse visits, with the next most common being risperidone misuse/abuse (5,804, 11%) and olanzapine misuse/abuse (4,528 cases, 9%), all figures similar to ours. 27

Quetiapine prescribing is common in the U.S. A 2013 IMS Health report showed that quetiapine was the most frequently prescribed SGA, with over 14 million dispensed prescriptions that year.^28^ Other studies support this, identifying a three-fold increase in prescribing over a decade,^29^ an observation likely driven by the increasing popularity of quetiapine use for “off-label” indications.^2^,^3^,^16^,^29^,^30^,^31^ These prescribing patterns may contribute to why quetiapine is the most commonly abused SGA in terms of absolute numbers of cases.

Aside from the public health concerns that emerge from these results, other outcomes of interest in this study were the medical consequences of quetiapine abuse. Clinical outcomes due to non-prescribed ingestions of quetiapine were recently described by a group of researchers who similarly used the NPDS, but in a smaller sample. Although their study combined cases characterized as “misuse” and “abuse,” the present study generally supports many of their findings regarding clinical outcomes. In this study, we confirmed that an ingestion of quetiapine for recreational purposes was likely to present symptomatic; 79.1% of cases with outcome data available described some degree of clinical effect, of which 26.6% were considered major or moderate effect. This finding is of particular importance to emergency physicians who will be caring for these patients.

According to our data, central nervous system (CNS) clinical effects will hallmark the quetiapine abuse patient presentation, as well as the presentation of any SGA abuse. SGAs treat both positive and negative symptoms of schizophrenia, and pharmacologically antagonize dopamine (D_2_) and serotonin (5HT_2a_) receptors.^32^ Thus, as expected, sedation was often observed in this study. Interestingly, certain severe CNS effects were significantly more common in the clozapine and olanzapine cohorts. While quetiapine, clozapine, and olanzapine are unique among SGAs in that they all have antagonistic activity at muscarinic (M_1_) receptors, olanzapine and clozapine are much more potent than quetiapine, which may be responsible for the increased incidence of agitation, confusion, coma, and hallucinations. In addition, clozapine is a known GABA-A receptor antagonist,^33^ and in previous data has been known to cause seizures at higher rates than other antipsychotics.^34^ Thus, the increased incidence of seizures seen for this particular medication in our study is not surprising.

Other than CNS effects, cardiovascular clinical effects were observed but were overall less common. Tachycardia was the most frequently observed cardiovascular clinical effect, followed by hypotension for most cohorts. While many SGAs cause adrenergic (α_1_) antagonism, which would typically lead to hypotension and reflex tachycardia, cardiovascular effects are often multi-factorial and in our data did not align with the varying degrees of α_1_-antagonism between drugs. The overall low rates of serious cardiovascular clinical effects suggest that hemodynamic instability is unlikely to be a key component of the presentation of SGA abuse, quetiapine or otherwise.

The intubation rate observed in this retrospective cohort of cases of quetiapine abuse was 1.4%, which represents a significant number of patients who may require airway management by emergency providers. The NPDS database does not specify reasons for intubation in each case but based on rates of clinical effects seen, CNS depression and/or severe agitation are the most likely indications. Studies characterizing quetiapine overdose identify much higher rates of intubation, suggesting a dose-dependent relationship regarding the need for intubation. One study found that 14 of 20 patients in their quetiapine overdose cohort of intensive care unit patients required mechanical ventilation.^20^ A larger retrospective review of 945 quetiapine overdose cases found an intubation rate of 16%.^18^ These findings should remind clinicians to have a high index of suspicion for acute respiratory failure in quetiapine abuse patients presenting after larger ingestions.

The rate of dystonia in the quetiapine abuse cohort was extremely low, with only 0.6% of cases manifesting this clinical effect. The pathophysiology of drug-induced dystonia is not wholly agreed upon. A commonly held theory is that a drug induces dystonia via dopamine (D_2_) antagonism in the nigrostriatal pathways of the basal ganglia, leading to excessive cholinergic input.^35^ This is supported by the presence of dystonic symptoms in patients with Parkinson’s disease as well as the observation that drugs with increased D_2_ antagonism cause dystonia that improves when antimuscarinic medications are administered. Considering the inherent antimuscarinic activity of quetiapine, olanzapine and clozapine, it is not surprising these three cohorts had the lowest rates of dystonia. This relative infrequency of dystonia in the quetiapine cohort could hypothetically contribute to quetiapine’s higher incidence of abuse, as dystonia is generally viewed as an undesirable side effect.

## LIMITATIONS

There are several limitations present in this study. The major limitation is its retrospective nature and the potential inaccuracy innate to the data available to the NPDS. Although highly trained poison center personnel collect NPDS data in real time, there was no means to verify data in this study, other than what was coded. NPDS data are at risk for certain misclassifications; however, this should be the same across all groups of SGAs and therefore mitigated. Cases can be incorrectly coded as single-substance ingestions when there were in fact co-ingestions, which could influence the reported clinical data. “Misuse” versus “abuse” could be interchangeably misclassified as well. Unfortunately, very limited data were collected regarding doses, which would have been helpful in understanding the clinical presentations of these cases. Prevalence of abuse is also likely underestimated in the present study due to the exclusion of co-ingestions and incomplete reporting to poison centers. (There is regional variability in poison center use as some poison centers charge hospitals for use and others preferentially use inpatient consulting toxicology services.) Again, these limitations however would hypothetically be similar for all medications included, so should not alter the conclusions regarding relative frequencies of SGA abuse.

## CONCLUSION

This study is a large retrospective cohort evaluating demographic features, clinical features, and the relative frequency of quetiapine abuse as it compares to other SGAs*.* According to these data, quetiapine is the most commonly abused SGA by a substantial margin. The findings of this study also confirm that most patients who present to the ED will be symptomatic and may require therapeutic interventions. It is important for emergency physicians to be aware of these findings, as they are likely to encounter this scenario in their clinical practice.

## Figures and Tables

**Table 1:** Clinical outcome definitions in the National Poison Data System^26^.

Medical outcome	Definition
Major effect	The patient exhibited signs or symptoms as a result of the exposure that were life-threatening or resulted in significant residual disability or disfigurement
Moderate effect	The patient exhibited signs or symptoms as a result of the exposure that were more pronounced, more prolonged, or more systemic in nature than minor symptoms. Usually, some form of treatment is indicated. Symptoms were not life-threatening, and the patient had no residual disability or disfigurement.
Minor effect	The patient developed some signs or symptoms as a result of the exposure, but they were minimally bothersome and generally resolved rapidly with no residual disability or disfigurement
Unable to follow	Insufficient follow up available
Not followed	Insufficient follow up available

**Table 2:** Patient demographics.

Demographics	Quetiapine (n = 2118)	All other SGAs (n = 1379)
Median age (years) (IQR)	17 (15 – 27)	18 (15 – 25)
Gender, male (%)	1313 (62.0%)	915 (66.4%)
Chronicity		
Acute	1685 (79.6%)	1044 (75.7%)
Acute on chronic	335 (15.8%)	260 (18.9%)
Chronic	32 (1.5%)	20 (1.5%)
Route of exposure		
Ingestion	1988 (93.8%)	1307 (94.5%)
Inhalation	120 (5.7%)	73 (5.3%)
Parenteral	16 (0.8%)	5 (0.4%)

All data provided as n (%) unless otherwise specified.

*SGA,* second-generation antipsychotics; *IQR,* inter-quartile range; if cases had multiple exposure routes coded, all were included.

**Table 3:** Disposition of patients coded as having intentionally abused second-generation antipsychotics (SGA).

Patient disposition	Quetiapine n = 2118	All other SGAs n = 1379	Aripiprazole n = 229	Clozapine n = 101	Olanzapine n = 246	Risperidone n =530	Ziprasidone n = 229
Treated and discharged	40.8%	39.4%	38.4%	23.4%	28.9%	44.3%	47.6%
Critical care admission	10.3%	9.3%	6.5%	22.8%	18.3%	5.8%	5.2%
Patient refused referral to hospital	7.8%	8.8%	10.4%	7.9%	9.3%	8.7%	6.1%
Psychiatric admission	7.2%	7.2%	9.6%	4.0%	8.5%	7.4%	5.2%
Non critical care admission	6.5%	6.6%	5.2%	14.8%	8.2%	6.2%	3.5%

All cases not included in table did not have available disposition data.

**Table 4:** Medical outcomes for each cohort.

Medical outcomes	Quetiapine n = 1446	All other SGAs n = 919	Aripiprazole n = 142	Clozapine n = 72	Olanzapine n = 167	Risperidone n = 361	Ziprasidone n = 149
Death	0.1%	0.1%	0	0	0	0.3%	0
Major outcome	2.0%	2.5%	0.7%	8.3%	5.4%	1.4%	1.3%
Moderate outcome	24.6%	37.6%	25.4%	50%	35.9%	44.0%	32.9%
Minor outcome + no effect	73.4%	76.8%	73.9%	41.3%	63.8%	54.1%	65.7%

*SGA,* second-generation antipsychotics

**Table 5:** Clinical effects seen with intentional abuse of second-generation antipsychotics.

Clinical effects	Quetiapine n = 1446	All other SGAs n = 919	Aripiprazole n = 142	Clozapine n = 72	Olanzapine n = 167	Risperidone n = 361	Ziprasidone n = 149
CNS effects							
Drowsy/lethargy	54.5%	39.4%	16.9%	62.5%	59.3%	31.6%	47.0%
Slurred speech	7.8%	6.4%	0.7%	16.7%	12.6%	4.2%	4.7%
Agitated/Irritable	5.5%	8.1%	3.5%	23.6%	16.2%	5.3%	3.4%
Dizziness/vertigo	5.0%	4.9%	4.9%	0	5.4%	3.9%	8.7%
Ataxia	4.4%	2.7%	0.7%	4.2%	7.2%	1.7%	2.0%
Confusion	4.2%	6.2%	3.5%	26.4%	11.4%	3.3%	0.7%
Hallucinations	1.6%	2.8%	0.7%	9.7%	4.8%	2.5%	0.7%
Coma	1.2%	1.6%	0	9.7%	3.0%	0.3%	1.3%
Seizures	0.8%	1.0%	1.4%	4.2%	1.8%	0.3%	0
Dystonia	0.6%	12.5%	12.0%	0	3.0%	19.1%	10.1%
Cardiovascular effects							
Tachycardia	22.9%	20.3%	14.1%	34.7%	19.2%	23.5%	12.1%
Hypotension	5.9%	3.0%	0	5.6%	1.8%	3.9%	4.7%
Syncope	1.8%	0.3%	0.7%	1.4%	0	0.3%	0
Conduction disturbance	1.2%	1.2%	1.4%	1.4%	0.6%	1.7%	0.7%
ECG changes	0.9%	0.5%	0	1.4%	0	0.3%	1.3%
Dysrhythmia	0.1%	0.1%	0	0	0	0.3%	0
Other effects							
Respiratory depression	1.0%	0.2%	0	0	1.2%	0	0
Elevated CK/rhabdomyolysis	0.4%	0.4%	0.7%	0	0.6%	0.6%	0
Respiratory arrest	0.1%	0.2%	0	0	0.6%	0.3%	0

*SGA,* second-generation antipsychotics; *CNS,* central nervous system; *ECG, electrocardiogram; CK,* creatine kinase

**Table 6:** Therapies provided to patients who intentionally abused second-generation antipsychotics (SGA).

Therapies	Quetiapine n = 1446	All other SGAs n = 919	Aripiprazole n = 142	Clozapine n = 72	Olanzapine n = 167	Risperidone n = 361	Ziprasidone n = 149
Intravenous fluids	24.5%	24.3%	14.8%	41.7%	31.1%	24.1%	18.1%
Charcoal	15.1%	15.2%	16.2%	11.1%	25.1%	12.7%	14.1%
Cathartics	4.6%	5.1%	5.6%	4.2%	9.0%	3.9%	4.7%
Oxygen	3.9%	3.0%	0.7%	8.3%	6.0%	2.2%	2.0%
Benzodiazepines	3.3%	6.0%	5.6%	12.5%	9.0%	4.4%	2.0%
Naloxone	2.4%	2.5%	0	8.3%	6.6%	0.8%	2.0%
Sedation	1.7%	0.1%	0	4.2%	3.6%	0	0
Intubation	1.4%	1.5%	0.7%	5.6%	4.2%	0.6%	0
Lavage	1.0%	1.1%	1.4%	0	3.0%	0.8%	0
Alkalinization	0.5%	0.2%	0	0	0.6%	0.8%	0
CPR	0.1%	0	0	0	0	0.3%	0
Physostigmine	0	0	0	0	0	0	0
Vasopressors	0	0	0	0	0	0.3%	0

*CPR,* cardiopulmonary resuscitation
